# Positive error-handling in TGfU-based basketball instruction: effects on tactical decision-making and the role of perceived classroom psychological safety

**DOI:** 10.3389/fpsyg.2026.1984425

**Published:** 2026-10-16

**Authors:** Shen Fan

**Affiliations:** Physical Education Teaching and Research Office, Department of Public Education, Anyang Vocational and Technical College, Anyang, China

**Keywords:** basketball, error climate, physical education pedagogy, psychological safety, tactical decision-making, teaching games for understanding

## Abstract

**Background:**

Basketball learning involves rapid decisions and visible mistakes that may carry interpersonal risk. This study examined whether positive error-handling within Teaching Games for Understanding (TGfU) improved tactical decision-making and whether improvements were associated with changes in perceived classroom psychological safety.

**Methods:**

Sixty students (30 male and 30 female) were individually randomized within gender strata before formation of two instructional classes, with one class per condition (*n* = 30 each). The same teacher delivered TGfU with positive error-handling (PEH) or neutral error-handling (NEH) over eight weeks. Tactical decision-making was assessed through standardized 3-on-3 games at pretest (T0), posttest (T1), and three-week retention (T2); psychological safety was assessed at T0 and T1. Passing, dribbling/driving, and shooting decisions were coded using the focused BALPAI rubric.

**Results:**

Exploratory student-level analyses showed a condition × time interaction for decision-making, *F*(1.583, 91.821) = 9.89, *p* < 0.001, ηp^2^ = 0.146. PEH increased from M = 1.951 (SD = 0.202) at T0 to 2.304 (0.258) at T1 and 2.295 (0.279) at T2; NEH increased from 1.902 (0.179) to 2.114 (0.251) and 2.087 (0.291). Psychological safety also showed a condition × time interaction, *F*(1, 58) = 11.07, *p* = 0.002, ηp^2^ = 0.160. PEH increased by 0.462 points, whereas the NEH increase of 0.129 points was not statistically significant. An exploratory within-subject mediation analysis in PEH yielded a model-implied indirect effect of ab = 0.160, 95% bootstrap CI [0.100, 0.221].

**Conclusion:**

The PEH class showed larger observed gains within the common TGfU curriculum, and increases in psychological safety covaried with decision-making improvements. These concurrent changes suggest a possible explanatory role for psychological safety but do not establish temporal or causal mediation. With one instructional class per condition, classroom-specific influences could not be separated from the condition effect, and inferential statistics were not adjusted for within-class dependence. Future studies should include multiple classes per condition, account for within-class dependence, and assess psychological safety before subsequent decision-making outcomes to examine the proposed explanatory process.

## 1 Introduction

Basketball is an open-skill invasion game in which successful performance depends on selecting an action that fits a rapidly changing configuration of teammates, opponents, space, time, and scoring opportunity. Passing, dribbling or driving, and shooting are not simply technical responses; each action is a decision coupled to information available in the game (de Souza et al., 2024). Ecological accounts therefore describe sport decisions as emerging from performer–environment relations rather than as detached choices made before movement. Recent work further emphasizes that individual and team decisions develop through continuous interaction with game-relevant information and constraints (Araújo et al., 2023, 2025). Representative practice tasks should therefore preserve the informational relations that guide perception, decision, and action in competition (Araújo et al., 2023; Manninen et al., 2025). For novice learners, the difficulty is not limited to executing a movement pattern: they must detect relevant opportunities, inhibit a superficially attractive option, and update a choice when the defensive configuration changes. An instructional approach that improves technique without improving choice quality leaves a central component of game competence underdeveloped.

Teaching Games for Understanding (TGfU) was developed as an alternative to technique-first instruction (Bunker and Thorpe, 1982). In TGfU, modified games and tactical problems precede or accompany focused skill practice, while questioning, reflection, and repeated play connect perception, decision, and action in representative contexts (González-Valero et al., 2024; Manninen et al., 2025). Subsequent work has also considered affective and social outcomes alongside tactical cognition within game-centered instruction (Ortiz et al., 2023; Pan et al., 2023; Arantes et al., 2025). Reviews of game-centered pedagogy consistently identify decision-making as a central intended outcome, while also emphasizing implementation quality and contextual variation (Ortiz et al., 2023; Yan et al., 2023; González-Valero et al., 2024; Manninen et al., 2025).

Quantitative syntheses provide cautious support for this rationale. Teaching-game interventions have shown advantages for decision-making, although the magnitude and consistency of these effects vary across studies and outcomes (Ortiz et al., 2023; González-Valero et al., 2024; Manninen et al., 2025). Randomized-trial and multilevel meta-analyses subsequently reported positive effects on game performance or decision-making, alongside null or variable effects for knowledge and some motor-skill outcomes (Ortiz et al., 2023; Manninen et al., 2025; González-Valero et al., 2024). In youth small-sided-game interventions, technical benefits are clearer than tactical evidence because some reviews found no eligible tactical-behavior outcomes (Clemente et al., 2021). Basketball-specific studies nevertheless show that tactical or TGfU-based curricula can improve game performance and related learning outcomes in authentic play (González-Espinosa et al., 2021; Pan et al., 2023). Together, this evidence supports TGfU as the common instructional foundation for both conditions, but it does not imply that every TGfU implementation produces the same effect (Morales-Belando et al., 2022).

Nevertheless, a game-centered task does not by itself guarantee psychologically productive exploration. Modified games make decisions public: a rushed shot, a pass to a marked teammate, or an unnecessary dribble can be observed immediately by peers and the teacher. When a learner expects embarrassment, blame, or status loss after an error, the rational short-term response may be to avoid the ball, choose a low-risk action, remain silent during tactical questioning, or copy a dominant peer. Such behavior reduces the very experimentation and explanation through which TGfU is expected to work. The pedagogical value of game representation may therefore depend partly on how the social environment treats unsuccessful decisions.

### 1.1 Errors as information and the teacher’s response

Errors can signal a mismatch between a learner’s current rule for action and the tactical information available in the environment. Their instructional value, however, is not automatic (Tulis and Dresel, 2025). Cognitive accounts indicate that error generation can support retention and higher-order learning when it is followed by diagnostic correction, comparison, and self-explanation (Zhang and Fiorella, 2023). Research on learning from errors and failure makes a related point: unsuccessful initial solutions can prepare learners to recognize the structure of a later explanation, although instructional support remains essential (Steuer et al., 2025b). Accordingly, the intervention is not designed to celebrate error for its own sake; it is designed to convert a visible error into information that can guide a revised action.

Positive error-handling (PEH) refers to a brief, structured post-error response in which the teacher uses learning-oriented language, preserves learner ownership of correction, allows additional thinking time, supports guided self-correction and comparison of alternatives, frames the error as useful learning information, and prevents ridicule (adapted from Soncini et al., 2021; Soncini et al., 2022; Zhang and Fiorella, 2023). In basketball instruction, the response also provides learners with an opportunity to implement the corrected decision in the same or an equivalent game situation, consistent with the situated and game-centered logic of TGfU (González-Valero et al., 2024; Manninen et al., 2025). This response begins only when a predefined tactical error remains identifiable after the common TGfU questions; PEH is not synonymous with TGfU questioning itself.

Classroom research shows that teachers’ verbal and nonverbal responses help determine whether mistakes become occasions for reasoning or cues for withdrawal (Soncini et al., 2022; Steuer et al., 2025a; Dresel et al., 2025). A constructive error climate includes respectful treatment, analysis of why a response was unsuccessful, opportunities for self-correction, and a shared understanding that mistakes can support learning (Soncini et al., 2021, 2022, 2024). Multilevel and longitudinal evidence links how mistakes are handled to student motivation, adaptive reactions, learning, and relationships with teachers, while also showing that effects can differ across the individual and classroom levels (Steuer et al., 2025a; Dresel et al., 2025). In contrast, responses that expose, ridicule, abruptly dismiss, or merely replace the learner’s contribution may close the learning episode before the underlying rule is reconstructed.

Soncini et al. (2021) provided experimental evidence that teacher error-handling can shape students’ perception of the classroom error climate. Importantly, both conditions used predetermined lesson questions; the manipulation concerned what the teacher did after an erroneous response. Their positive protocol combined time to reconsider, prompts supporting self-correction, discussion of alternative solutions, an explicit learning-oriented interpretation of errors, and protection from negative peer reactions. Later evidence linked positive error climate to learning outcomes through more adaptive affective, motivational, and cognitive-behavioral reactions to errors (Soncini et al., 2022). Research on learning from errors points in the same direction: active exploration supported by metacognitive and emotion-regulation processes can improve adaptive learning and transfer (Zhang and Fiorella, 2023; Steuer et al., 2025b). The present study therefore nests an error-response manipulation within a common inquiry-oriented basketball lesson rather than treating questioning itself as unique to PEH, while not claiming that the basketball adaptation is already a validated package.

The comparison condition is neutral error-handling (NEH). Before any condition split, the teacher uses the same TGfU questions in both classes. If an eligible tactical error remains evident, the NEH teacher respectfully identifies the less functional choice, states the relevant cue and a preferable option, and gives the learner an opportunity to replay the situation. This is an educationally credible active comparison because learning-from-error accounts emphasize that diagnostic corrective information, explanation, and an opportunity to revise can support learning (Zhang and Fiorella, 2023; Narciss and Alemdag, 2025). NEH does not add a second learner-owned reconstruction period, invite peer comparison, or explicitly frame the error as a learning resource. Ridicule is prohibited in both conditions. Thus, the study compares two post-error response scripts nested within the same TGfU inquiry structure; it does not compare TGfU questioning with an absence of questioning or with a separate direct-instruction curriculum.

### 1.2 Perceived classroom psychological safety as a mechanism

Psychological safety refers to a state in which interpersonal risk is reduced and individuals feel able to speak up, seek help, acknowledge uncertainty, and discuss failure (Edmondson and Bransby, 2023; Vella et al., 2024; Walton et al., 2024). It is distinct from general comfort, trust, or the absence of demanding standards; rather, it concerns the perceived interpersonal consequences of these behaviors (Edmondson and Bransby, 2023). Reviews link psychological safety to learning behavior, information sharing, engagement, and performance, although these relationships vary across levels and contexts (Edmondson and Bransby, 2023; Vella et al., 2024). In a basketball class, the relevant risks include explaining a failed decision, proposing an unconventional solution, and continuing to seek the ball after a visible mistake.

Sport research supports the relevance of the construct but also requires conceptual restraint (Taylor et al., 2022). Psychological safety in sport has been described as a continuous group-level property perceived and reported by individuals, with heterogeneous definitions and measures across studies (Vella et al., 2024). Recent research links psychologically safe environments with team functioning, well-being, voice, and coach–athlete relationship quality (Jowett et al., 2023; Vella et al., 2024; Hurst and Kavussanu, 2026). These studies do not establish the present classroom mediation model, but they show that interpersonal safety is relevant in embodied, performance-visible group settings.

Teacher error-handling should influence psychological safety because it provides repeated public information about the interpersonal consequences of being wrong. Inclusive leader behavior—words and actions that invite and appreciate contributions—has been associated with psychological safety and improvement-oriented participation in learning-intensive settings (Edmondson and Bransby, 2023). Supportive interpersonal relationships are likewise relevant to psychological safety in sport settings (Jowett et al., 2023; Vella et al., 2024). A teacher who waits, invites revision, and blocks ridicule may therefore communicate that an imperfect action does not threaten belonging. The same behavior can make tactical discussion diagnostically useful because students can reveal what they perceived and compare their rule for action with alternatives.

If positive error-handling increases perceived safety, safer learners should participate more fully in TGfU learning cycles. They may verbalize uncertainty during questioning, attend to feedback instead of defending self-image, attempt a revised decision, and remain involved after failure. Reviews suggest that learning behavior can be one pathway linking psychological safety with effectiveness rather than psychological safety operating as a direct performance treatment (Edmondson and Bransby, 2023). In higher education, however, a brief intervention targeting psychological safety and trust did not produce consistent change, including among physical education students (Senden et al., 2023). Psychological safety is therefore positioned as a theoretically plausible explanatory mechanism rather than an assumed consequence of repeated exposure to positive error-handling.

### 1.3 The present study

Existing research supports game-centered instruction for decision-making and constructive error climates for adaptive learning, but these literatures have rarely been integrated in basketball instruction. The present study holds the TGfU curriculum, initial tactical questions, and opportunity to replay constant; the instructional conditions branch only after a predefined tactical decision error remains evident. A focused BALPAI adaptation is used to preserve an authentic game-based outcome while keeping observation feasible: only decisions associated with passing, dribbling/driving, and shooting are coded. The study also adds a three-week retention test to distinguish immediate performance change from short-term stability. The intended contribution is therefore a mechanism-focused test of whether the teacher’s post-error response changes how learners use an otherwise common TGfU curriculum.

The study tests the following hypotheses:

H1. Tactical decision-making will show a significant condition × time interaction, with greater improvement in the PEH condition than in the NEH condition.

H2. Perceived classroom psychological safety will show a significant condition × time interaction, with a greater pre-post increase in the PEH condition than in the NEH condition.

H3. Within the PEH condition, greater increases in perceived classroom psychological safety will be associated with greater pre-post improvements in tactical decision-making.

## 2 Materials and methods

### 2.1 Design

This study used a two-arm, gender-stratified randomized instructional design with repeated measurements at pretest (T0), immediate posttest (T1), and a three-week retention test (T2). Individual random allocation was used to reduce systematic baseline differences between conditions (Heindel et al., 2022), while stratification by gender was used to balance gender composition across the two instructional groups, consistent with the use of stratification to balance selected baseline characteristics (Bruce et al., 2022). The two-arm design compared PEH with an active NEH comparator while holding the TGfU curriculum, instructional tasks, and instructional duration constant. Repeated measurements were used to examine immediate change and short-term retention. Reporting of participant allocation, follow-up, and between-condition comparisons was informed by CONSORT 2025 recommendations for parallel-group randomized trials (Hopewell et al., 2025). Sixty students (30 male and 30 female) were recruited into a common eligible sample. Before the instructional classes were formed, participants were stratified by gender and randomly allocated to two newly formed classes (n = 30 per class), yielding 15 male and 15 female students in each class. Within each gender stratum, allocation to PEH or NEH was performed in a 1:1 ratio using a computer-generated random sequence. One class received TGfU combined with positive error-handling (PEH), whereas the other received the matched TGfU curriculum combined with neutral error-handling (NEH). The same teacher delivered both conditions. The TGfU curriculum, teaching tasks, core tactical questions, instructional duration, and assessment schedule were identical; only the condition-specific response after an unresolved eligible tactical error differed. Students were informed about their own instructional activities but were not told the content of the alternative condition. Perceived classroom psychological safety was assessed at T0 and T1, and tactical decision-making at T0, T1, and T2 (see Figure 1).

**Figure 1:**
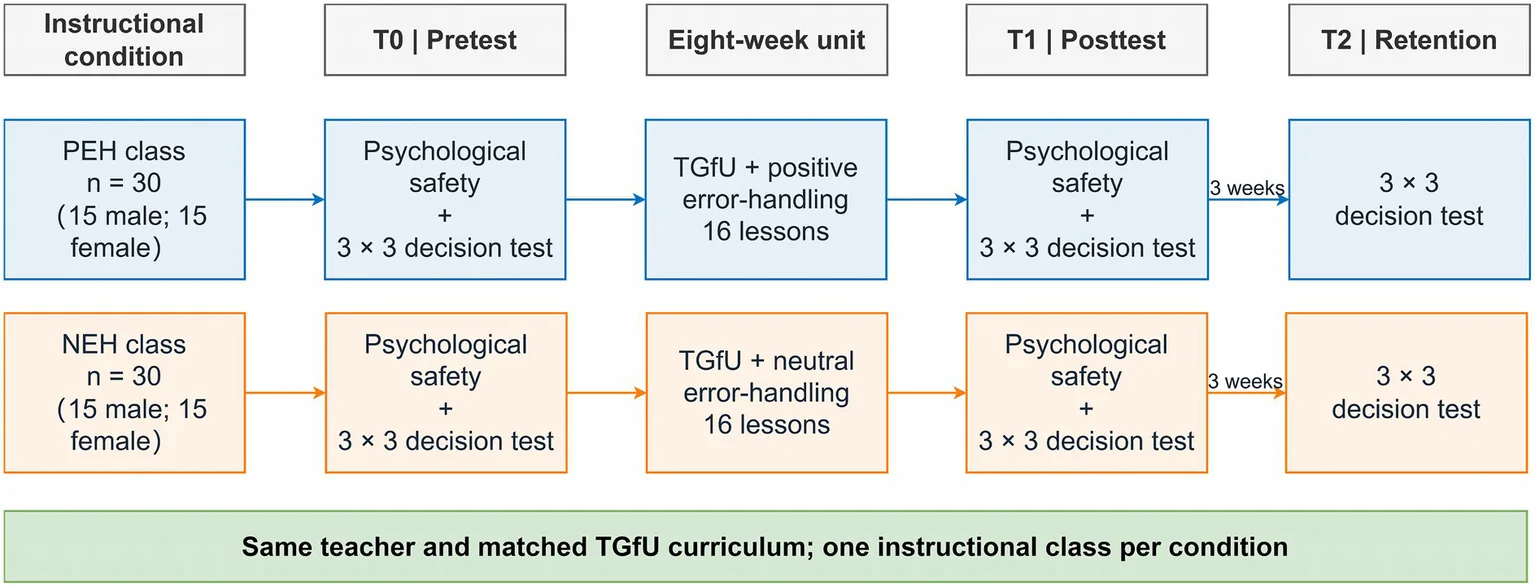
Study design.

The decision-making design was therefore 2 (condition: PEH, NEH) × 3 (time: T0, T1, T2), whereas the psychological-safety design was 2 × 2 (T0, T1). Randomization occurred at the individual level before class formation, whereas the intervention was delivered at the classroom level, with one instructional class per condition. Students within the same class may share peer norms, interactions, and other contextual influences. Consequently, classroom-specific influences could not be estimated separately from the condition contrast. Methodological guidance on cluster-randomized trials highlights the importance of cluster replication and accounting for within-cluster dependence (Hemming and Taljaard, 2023). Although the present study randomized individual students rather than classes, dependence arising during classroom instruction remains relevant. Accordingly, student-level inferential findings are interpreted as exploratory, and future studies should include multiple instructional classes per condition to support estimation of classroom-level variation.

### 2.2 Participants and setting

Participants were recruited using volunteer, non-probability sampling from non-physical-education majors enrolled in a basketball course at Anyang Vocational and Technical College, Anyang, China (Stratton, 2023). Recruitment information specifying the study and eligibility requirements was distributed before study enrollment. Sixty-five students volunteered and underwent eligibility screening. Eligibility required: (a) enrollment in a non-physical-education major; (b) passing a basic health and physical-readiness screening for safe participation in basketball activities; (c) no current injury preventing participation; and (d) no substantial competitive basketball experience, because the intervention targeted novice or low-experience learners. Five students did not pass the physical-readiness screening and were excluded, leaving 60 eligible participants (30 male and 30 female). After recruitment and screening, participants were stratified by gender and randomly allocated in a 1:1 ratio to PEH or NEH using a computer-generated sequence, resulting in two newly formed classes of 30 students, each comprising 15 male and 15 female students. Recruitment, eligibility screening, allocation, and participant flow were reported in accordance with relevant CONSORT 2025 recommendations (Hopewell et al., 2025).

The study was reviewed and approved by the Academic Ethics Committee of Anyang Vocational and Technical College (approval No. 202602003). All participants provided written informed consent before participation. Participation was voluntary, participants could withdraw at any time without penalty or any effect on course grades, and each participant received a small gift of appreciation that was not contingent on performance or study outcomes. The procedures followed the Declaration of Helsinki.

### 2.3 Common TGfU curriculum

Both classes completed 16 lessons over 8 weeks (two 90-min lessons per week) delivered by the same trained instructor. The curricular content, court space, equipment, modified-game constraints, tactical questions, practice volume, and assessment schedule were identical across conditions. The unit progressed through three recurring offensive problems: (a) maintaining possession and creating a passing lane, (b) attacking space through dribbling or driving, and (c) creating and selecting a high-quality shooting opportunity.

Each lesson followed the same TGfU teaching and learning sequence: (1) a warm-up with ball activities; (2) an initial modified game, such as 3 × 3 play, that presented the tactical problem; (3) guided questioning and feedback about relevant cues, available options, and reasons for decisions; (4) focused skill practice to implement tactical solutions; (5) further modified game play under adjusted constraints to apply tactical decisions and skills; and (6) a cool-down and learning reflection. The common lesson structure and the elements held constant across conditions are summarized in Table 1. This lesson structure is consistent with the emphasis on modified games that preserve tactical structure and guided inquiry that supports tactical awareness and decision-making in TGfU and game-based approaches (González-Valero et al., 2024; Wang et al., 2024; Manninen et al., 2025). At every planned feedback episode, before any condition split, the teacher used the same three core prompts in both classes: What did you see? What options were available? Why did you choose that action? Adequate decisions received the same brief confirmation. Only when the action met the tactical-error criterion and remained unresolved after these common questions did the teacher begin the PEH or NEH post-error script. Feedback opportunities were planned around three recurring action categories—passing, dribbling/driving, and shooting. Multiple eligible feedback episodes could occur within these categories as relevant events arose naturally during play.

**Table 1:** Common TGfU lesson structure across instructional conditions.

Teaching and learning phase	Approx. time	Learning focus	Elements held constant across conditions
Warm-up with ball activities	10 min	Prepare students physically for basketball activities	Tasks, equipment, and work-to-rest ratio
Modified game play (tactical awareness)	15 min	Recognize the lesson’s tactical problem through game play	Game format, rules, and numerical balance
Guided questioning and feedback (tactical decision-making)	15 min	Identify relevant cues, discuss available options, and explain decisions	Same tactical problem and core questions; feedback opportunities organized around passing, dribbling/driving, and shooting; only the post-error response differed
Focused skill practice (skill execution)	20 min	Develop the basketball skills needed to implement tactical solutions	Practice repetitions and task difficulty
Modified game play (application of tactical decisions and skills)	25 min	Apply and refine tactical decisions and skills under adjusted game constraints	Task constraints, opponent level, and teacher observation
Cool-down and learning reflection	5 min	Consolidate understanding of the lesson’s tactical principle	Reflection prompt, except for condition-specific error-handling language

Terms in parentheses indicate the primary learning focus of each phase; these learning processes may occur across multiple phases.

### 2.4 Condition-specific error-handling

A tactical error was operationally defined as a pass, dribble/drive, or shot for which the game configuration contained a clearly more functional alternative according to the focused BALPAI criteria described below. Technical execution failure alone (e.g., an accurate decision followed by a missed open shot) did not trigger the tactical error protocol. This distinction prevented the teacher from treating unsuccessful outcome as synonymous with poor decision.

In the PEH condition, after the common TGfU questions had been delivered and an eligible error remained, the teacher used a five-component sequence adapted from experimental classroom error-handling research (Soncini et al., 2021): acknowledge the error in neutral, learning-oriented language; retain the original learner’s turn and provide at least 5 sec of additional wait time; use a cue to support self-correction rather than immediately supply the answer; invite one or two alternative solutions from peers; and explicitly frame the error as useful information while preventing ridicule or dismissive reactions. The learner then replayed the situation or encountered an equivalent restart.

In the NEH condition, the same common TGfU questions preceded the condition-specific response. If the eligible error remained, the teacher neutrally identified the less functional choice, explained the tactically relevant cue, stated a preferred option, and provided a replay opportunity. The teacher remained respectful and intervened against overt ridicule in both conditions for ethical reasons. NEH did not add the PEH-specific learner-owned wait period, invite peer alternatives, or explicitly normalize the error as a learning resource. These shared and condition-specific elements are summarized in Table 2. Feedback-window duration was standardized as closely as practicable across conditions; any remaining NEH time was used for a brief teacher explanation, demonstration, or additional replay.

**Table 2:** Contrast between positive error-handling and neutral error-handling.

Element	Positive error-handling (PEH)	Neutral error-handling (NEH)
Common TGfU questioning (identical)	Same preplanned prompts: What did you see? What options were available? Why did you choose that action?	Same preplanned prompts: What did you see? What options were available? Why did you choose that action?
Trigger for condition-specific script	Begins only after a predefined tactical error remains evident following the common questions.	Begins only after a predefined tactical error remains evident following the common questions.
First post-error response and ownership	Learning-oriented acknowledgment; original learner retains the turn, receives ≥5 s, and is cued to revise.	Neutral identification; teacher explains the relevant cue and supplies a preferred option.
Peer participation and meaning	Peers may compare alternatives; error is explicitly framed as information for learning.	No additional peer comparison; error is treated as a choice to correct without learning-oriented framing.
Ethical floor	Teacher proactively supports respectful participation and prevents or redirects ridicule.	Teacher remains respectful and prevents overt ridicule; no humiliating response is permitted.
Retry and instructional dose	Immediate replay; question count and retry opportunity were standardized; feedback-window duration was standardized as closely as practicable across conditions.	Immediate replay; question count and retry opportunity were standardized; feedback-window duration was standardized as closely as practicable across conditions.
Example after a contested shot with an open teammate	Common prompt first. If unresolved: ‘This gives us useful information. Take 5 sec, check the help defender, and revise your choice. What alternative do you see?’	Common prompt first. If unresolved: ‘The help defender closed and the corner teammate was open. The preferred choice is the pass. Let us replay it.’

### 2.5 Instructor preparation and implementation fidelity

As illustrated in Figure 2, the instructor completed a two-hour workshop using a written manual, video examples, and role-play. Training separated two layers explicitly: a common TGfU prompt card, identical in both classes, and condition-specific post-error response cards for PEH and NEH. To reduce carryover, the response cards and teaching logs were condition-specific, whereas the tactical-question list was identical. The order in which classes were taught was alternated where scheduling permitted. The instructor recorded the occurrence, target action, common questions delivered, post-error script used, and approximate duration of each planned episode.

**Figure 2:**
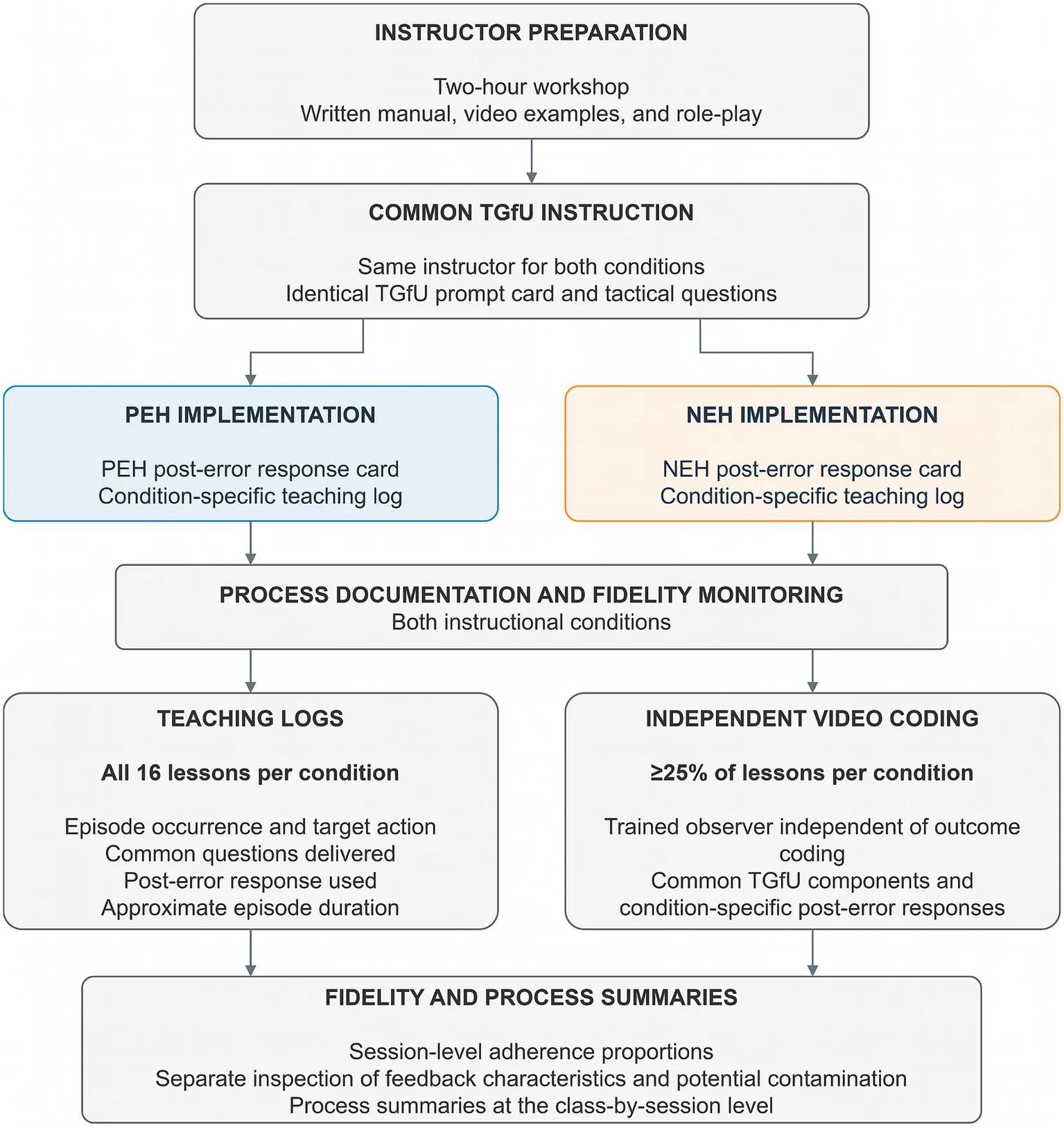
Instructor preparation and implementation fidelity monitoring procedures.

At least 25% of lessons in each condition were independently coded from video by a trained observer who was not involved in outcome coding. The fidelity observer was not informed of the study hypotheses or the formal class-to-condition assignment. However, because the instructional behaviors being coded were visible in the recordings, complete blinding to condition could not be guaranteed. All 16 sessions per condition contributed class-by-session process records. Fidelity coding covered both the common TGfU layer (modified-game task, core questions, episode timing, practice dose, and replay opportunity) and the condition-specific post-error layer (PEH components or the corresponding NEH response, feedback duration, and potential contamination events). A session-level adherence proportion was calculated for each lesson, while contamination indicators and feedback characteristics were inspected separately. Process summaries were treated as class-by-session observations rather than as 60 independent student observations.

### 2.6 Measures

#### 2.6.1 Participant characteristics

At baseline, participants reported age, gender, prior basketball experience, organized sport participation, and recent injury. Attendance was recorded for every lesson. Prior experience and attendance were described.

#### 2.6.2 Perceived classroom psychological safety

Perceived classroom psychological safety was measured with a context-adapted version of Edmondson's (1999) seven-item Team Psychological Safety Scale. The referent was changed from the work team to “this basketball class,” and examples were framed around asking for help, admitting uncertainty, and making mistakes during learning. Responses used a five-point scale from 1 (strongly disagree) to 5 (strongly agree). Negatively worded items were reverse-coded and averaged so that higher scores indicated greater perceived safety.

Because the intervention occurred in a Chinese-language educational setting, two bilingual researchers translated and back-translated the items, and an expert panel evaluated semantic and contextual equivalence. A small pilot outside the analytic sample checked comprehension. Structural validity was further examined separately at T0 and T1 using exploratory factor analysis (EFA) and a theory-specified one-factor confirmatory factor analysis (CFA). Internal consistency was reported using McDonald’s ω and Cronbach’s α at each wave. The construct was analyzed as an individual perception; with only two classes, aggregation to a class-level score and multilevel construct validation were not defensible.

#### 2.6.3 Tactical decision-making in standardized 3 × 3 play

Tactical decision-making was assessed from video of standardized 3 × 3 half-court games. The 3 × 3 half-court format was selected over full-court 5 × 5 play to provide novice or low-experience learners with fewer players to monitor while preserving teammate cooperation and defensive opposition, consistent with TGfU principles of game modification (González-Valero et al., 2024; de Souza et al., 2025). It matched the targeted passing, dribbling/driving, and shooting decisions without introducing full-court transition demands, while also facilitating consistent video coverage. The assessment therefore characterized decision-making in this format.

The broader Game Performance Assessment Instrument established a precedent for observing decision-making and skill execution in authentic play (Oslin et al., 1998). More recent reviews confirm that decision-making and skill execution remain among the most frequently assessed GPAI components in physical education, while also cautioning that composite indices may obscure the specific nature of students’ learning (Manso-Lorenzo et al., 2024). Methodological work has likewise raised concerns regarding opaque composite indices, nonlinear ratios, and insufficient observer-reliability reporting (Memmert and Harvey, 2008). The Basketball Learning and Performance Assessment Instrument (BALPAI) supplies basketball-specific categorical criteria for decision-making, execution, and efficacy across 11 actions, using scores of 1 (inadequate), 2 (neutral), and 3 (adequate) (Ibáñez et al., 2019). To keep observation feasible and align the outcome with the instructional unit, the present study retained only three on-ball offensive decisions: passing, dribbling/driving, and shooting. This focused index was treated as a study-specific adaptation rather than as a newly validated version of the full BALPAI.

Two further studies informed this decision. A video-based basketball assessment differentiated decision skill in youth players and demonstrated the feasibility of standardized visual stimuli, although it measured an off-court perceptual-cognitive response rather than choice during live play (Rösch et al., 2021). An external evaluation of BALPAI in competitive male youth basketball found limited discrimination by selection status and playing position, cautioning against assuming that the instrument functions identically across populations and purposes (Rösch et al., 2022). The current novice classroom study therefore retained the published action categories and decision-rating logic, reported action-specific scores and event counts separately in Supplementary Table S2, and treated the combined focused score as study-specific.

At each test, students played one eight-minute game divided into two four-minute periods with a two-minute interval. Stable three-player testing units and comparable opponents were used across waves where attendance permitted. Court dimensions, ball, basket height, possession rules, camera position, and verbal instructions remained constant. The assessing instructor provided no tactical feedback. Every eligible pass, dribble/drive initiation, and shot was coded. A participant’s primary decision score was calculated as the arithmetic mean of all eligible scored on-ball action events at that wave. Action-specific means were calculated separately for passing, dribbling/driving, and shooting. Participants with fewer eligible events were retained in the full-sample analyses, with their scores calculated from their available events. Each participant contributed one participant-level score per outcome at each wave, without weighting participants by their event counts. Periods without an eligible on-ball action did not enter the numerator or denominator and were not assigned an inadequate-decision rating. These scores therefore represented the quality of observed decisions rather than the frequency of participation. Event counts referred to eligible coded decision events rather than every physical contact with the ball.

The operational criteria used to classify passing, dribbling/driving, and shooting decisions are summarized in Table 3.

**Table 3:** Focused BALPAI decision-coding rubric.

Action	3 = adequate	2 = neutral/ambiguous	1 = inadequate
Pass	Passes to an available teammate in a position that maintains possession or improves attacking advantage.	The receiver is available but the pass offers limited advantage, or relevant cues support more than one reasonable option.	Passes to a closely marked or disadvantaged teammate when a clearly safer or more advantageous option is available.
Dribble/drive	Attacks exploitable space when no clearly better passing option is available, or uses the dribble to create a functional advantage.	Either defensive pressure is present or a clearly available teammate exists, but not both; the option is defensible but not clearly best.	Dribbles into defensive pressure while a clearly available teammate is positioned to receive and advance play.
Shot	Shoots without clear disruptive pressure when the shooter’s opportunity is at least as favorable as teammates’ available opportunities.	Either meaningful defensive pressure is present or a teammate has a clearly more favorable opportunity, but not both.	Shoots under meaningful pressure while a teammate has a clearly more favorable opportunity.

The decision context immediately preceding action initiation determined the rating; the eventual technical outcome did not alter the decision score. For example, an open, tactically justified shot that missed remained an adequate decision. Ambiguous clips, occluded views, and actions occurring after a stoppage were marked unscorable and excluded. Coding examples were assembled in a study manual and reviewed by 3 basketball-pedagogy experts before data collection.

Two coders blinded to condition, test wave, and study hypotheses completed training using non-study video. A stratified subsample of 16 participants contributed 48 participant-by-wave videos that were independently double-coded, yielding 807 paired action-event ratings for reliability analysis. Agreement for the 1–3 ordinal action ratings was quantified at the event level using linear weighted Cohen’s κ. As a supplementary absolute-agreement index, a two-way random-effects, absolute-agreement, single-measure intraclass correlation coefficient [ICC(2,1)] was calculated using the same paired event-level PrimaryScore and Rater2Score ratings. Thus, both reliability coefficients used the action event, rather than the participant mean, as the unit of analysis. The ICC form, unit, and intended interpretation were specified because different ICC formulations answer different reliability questions (Shrout and Fleiss, 1979; Koo and Li, 2016). Disagreements in the double-coded set were resolved only after reliability was calculated; the remaining videos were divided between the two coders.

#### 2.6.4 Manipulation check

A brief five-item perceived error-handling check, derived from the five PEH components, was administered after the final lesson. Items assessed whether the teacher allowed additional time to think after an error, retained learner ownership of correction, discussed alternatives, treated mistakes as useful, and prevented negative reactions. Responses ranged from 1 (never) to 5 (consistently). This researcher-developed measure was used only to verify post-error condition separation and did not replace the psychological-safety measure or enter the primary mediation model. Separate observer items verified that the common TGfU question content, frequency, and timing were matched across classes.

### 2.7 Procedure

Preparation: the manual was finalized, expert review and piloting were completed, and the instructor and observers were trained.

Pretest (T0): consent and baseline characteristics were obtained; the psychological-safety scale was administered; and standardized 3 × 3 games were recorded.

Intervention: 16 matched TGfU lessons were delivered over eight weeks; attendance, teacher logs, and fidelity video were collected.

Posttest (T1): within seven days of the final lesson, the psychological-safety scale and standardized 3 × 3 games were repeated; the manipulation check was administered.

Retention (T2): Three weeks after T1, the standardized 3 × 3 game test was repeated. Participants were instructed to refrain from basketball-related practice and games during the three-week retention interval. Compliance was not systematically monitored, and adherence to this instruction could not be verified.

Blind coding and analysis: video was de-identified, file order was randomized, reliability checks were completed, the database was locked, and the analyses were conducted.

### 2.8 Statistical analysis

Analyses were conducted in IBM SPSS Statistics with two-sided α = 0.05. Descriptive statistics and distributions were inspected before inferential testing. Baseline characteristics were summarized by condition; standardized mean differences (SMDs) were emphasized because modest samples can show chance imbalance after randomization. Missing item responses followed the scoring rule of the psychological-safety scale. All 60 participants contributed complete outcome data at T0, T1, and T2.

Because each instructional condition was implemented in only one class, the student-level ANOVAs and the exploratory within-subject mediation analysis in the PEH condition were treated as exploratory. The reported inferential statistics do not account for potential within-class dependence and were not adjusted for class-level clustering. With only two instructional classes, between-class variance cannot be estimated reliably or separated from condition, precluding a defensible multilevel or cluster-robust analysis.

For tactical decision-making, a mixed repeated-measures analysis of variance tested condition (PEH vs. NEH) × time (T0, T1, T2). Mauchly’s test assessed sphericity, and Greenhouse–Geisser-adjusted degrees of freedom were reported when the assumption was violated. A significant interaction was followed by simple-effects comparisons: between-condition contrasts were examined at each time point, and within-condition pairwise comparisons across T0, T1, and T2 were Bonferroni-adjusted. Partial eta squared (ηp^2^), mean differences, standard errors, and 95% confidence intervals were reported. As a sensitivity analysis, ANCOVA was used to examine whether adjustment for baseline decision score altered the between-condition differences at T1 and T2; additional models also adjusted for age and gender. Action-specific decision scores for passing, dribbling/driving, and shooting were examined using separate 2 × 3 condition × time mixed ANOVAs. An additional exploratory sensitivity analysis conducted during revision repeated each action-specific model after excluding participants with fewer than three eligible events for that action at any assessment wave. The three-event criterion was an exploratory threshold rather than a prespecified or validated reliability cutoff, and the full-sample analyses remained the primary analyses.

For perceived psychological safety, a 2 (condition: PEH, NEH) × 2 (time: T0, T1) mixed analysis of variance tested the interaction, followed by paired pre-post contrasts within each condition. Fidelity indices were summarized descriptively because the process observations were recorded at the class-by-session level. The student-reported manipulation check was used as a descriptive validation of perceived condition separation and was not treated as a primary outcome.

An exploratory within-subject mediation analysis was conducted within the PEH condition using MEMORE 2.1, Model 1, in SPSS (Montoya and Hayes, 2017). Within the PEH condition, the independent contrast was time (T1 vs. T0), the mediator was perceived psychological safety measured at T0 and T1, and the outcome was tactical decision-making measured at T0 and T1. The indirect effect was estimated from the within-person change model using 5,000 bootstrap resamples. Total and direct effects were also reported. Because psychological safety and tactical decision-making were assessed over the same T0-T1 interval, this analysis examines concurrent within-person covariation between their pre-post changes and does not establish temporal precedence or causal mediation.

## 3 Results

### 3.1 Participant flow and baseline characteristics

All 60 enrolled students completed T0, T1, and T2, yielding complete-case analyses of n = 30 in PEH and n = 30 in NEH. Gender was balanced by design, with 15 male and 15 female students in each condition. Attendance averaged 94.6% (SD = 5.1%) in PEH and 90.0% (SD = 7.4%) in NEH. Baseline characteristics are reported in Table 4. Small chance imbalances were observed in age (SMD = 0.331) and baseline decision-making (SMD = 0.255), whereas prior basketball experience, organized sport participation, and baseline psychological safety were closely balanced.

**Table 4:** Participant characteristics at baseline.

Characteristic	PEH (*n* = 30)	NEH (n = 30)	Standardized difference
Age, years, M (SD)	20.43 (1.38)	20.01 (1.23)	0.331
Prior basketball experience, months, M (SD)	2.77 (3.21)	2.80 (2.93)	−0.011
Organized sport participation, *n* (%)	3 (10.0%)	4 (13.3%)	−0.104
Baseline psychological safety (1–5), M (SD)	2.971 (0.488)	2.948 (0.667)	0.041
Baseline decision score (1–3), M (SD)	1.951 (0.202)	1.902 (0.179)	0.255

### 3.2 Measurement reliability, implementation fidelity, and manipulation check

The event-level file contained 3,039 unique action events from all 60 participants, covering 540 student × time × action combinations. Final Score followed the prespecified adjudication rule: 2,232 single-coded events used the primary score, 665 double-coded agreements used the common score, and 142 disagreements were resolved by third-rater adjudication. Based on the same 807 paired event-level PrimaryScore and Rater2Score ratings, linear weighted Cohen’s κ was 0.779 and the absolute-agreement ICC(2,1) was 0.838. Psychological-safety internal consistency was α = 0.875 and ω = 0.879 at T0, and α = 0.916 and ω = 0.917 at T1.

The adapted psychological-safety scale also provided support for a one-factor structure at both waves. EFA loadings ranged from 0.612 to 0.860 at T0 and from 0.723 to 0.855 at T1. KMO values were 0.860 and 0.897, respectively, and Bartlett’s tests were significant at both waves (*p* < 0.001). Parallel analysis supported retention of one factor at both T0 and T1. In the one-factor CFA, standardized loadings ranged from 0.673 to 0.922 at T0 and from 0.760 to 0.906 at T1. The WLSMV models yielded scaled CFI/TLI values of 0.992/0.988 at T0 and 0.993/0.990 at T1, RMSEA values of 0.075 and 0.091, and SRMR values of 0.061 and 0.048, respectively.

Across the 32 class-by-session process records, mean session-level adherence was 0.892 (SD = 0.05) in PEH and 0.879 (SD = 0.03) in NEH. The mean number of coded feedback episodes per session was 19.19 in PEH and 17.13 in NEH, and mean feedback-window durations were 42.04 s and 37.91 s, respectively.

The five-item manipulation check showed good internal consistency (α = 0.94). Students in the PEH condition reported greater exposure to the five positive error-handling components (M = 4.267, SD = 0.534) than those in the NEH condition (M = 2.520, SD = 0.472), yielding a descriptive difference of 1.747 points.

### 3.3 Tactical decision-making

Mauchly’s test indicated that the assumption of sphericity was violated, W = 0.737, χ^2^(2) = 17.420, *p* < 0.001, with a Greenhouse–Geisser epsilon of 0.792. The Greenhouse–Geisser-corrected condition × time interaction for the overall decision score was significant, *F*(1.583, 91.821) = 9.89, *p* < 0.001, ηp^2^ = 0.146. This condition × time pattern is interpreted as exploratory given the one-class-per-condition implementation. The conditions did not differ significantly at T0 (mean difference = 0.049, SE = 0.049, 95% CI [−0.050, 0.147], *p* = 0.327). PEH scored significantly higher than NEH at T1 (mean difference = 0.190, SE = 0.066, 95% CI [0.059, 0.322], *p* = 0.005) and T2 (mean difference = 0.208, SE = 0.074, 95% CI [0.060, 0.355], *p* = 0.007).

ANCOVA sensitivity analyses adjusting for baseline decision score yielded the same substantive conclusion at T1 and T2; the adjusted PEH–NEH differences remained statistically significant, and additional adjustment for age and gender produced nearly identical estimates (Supplementary Table S1).

In Bonferroni-adjusted within-condition comparisons, PEH increased by 0.354 points from T0 to T1 (SE = 0.026, 95% CI [0.289, 0.418], *p* < 0.001) and by 0.345 points from T0 to T2 (SE = 0.034, 95% CI [0.261, 0.428], *p* < 0.001); the 0.009-point decrease from T1 to T2 was not significant (SE = 0.022, 95% CI [−0.045, 0.063], *p* = 1.000). NEH increased by 0.212 points from T0 to T1 (SE = 0.026, 95% CI [0.147, 0.276], *p* < 0.001) and by 0.185 points from T0 to T2 (SE = 0.034, 95% CI [0.102, 0.269], *p* < 0.001); the 0.026-point decrease from T1 to T2 was not significant (SE = 0.022, 95% CI [−0.027, 0.080], *p* = 0.694). Thus, both conditions retained their posttest gains, with larger observed gains in PEH at posttest and retention (Figure 3; Table 5). Action-specific analyses showed significant condition × time interactions for passing and shooting, but not for dribbling/driving (Supplementary Table S2).

**Figure 3:**
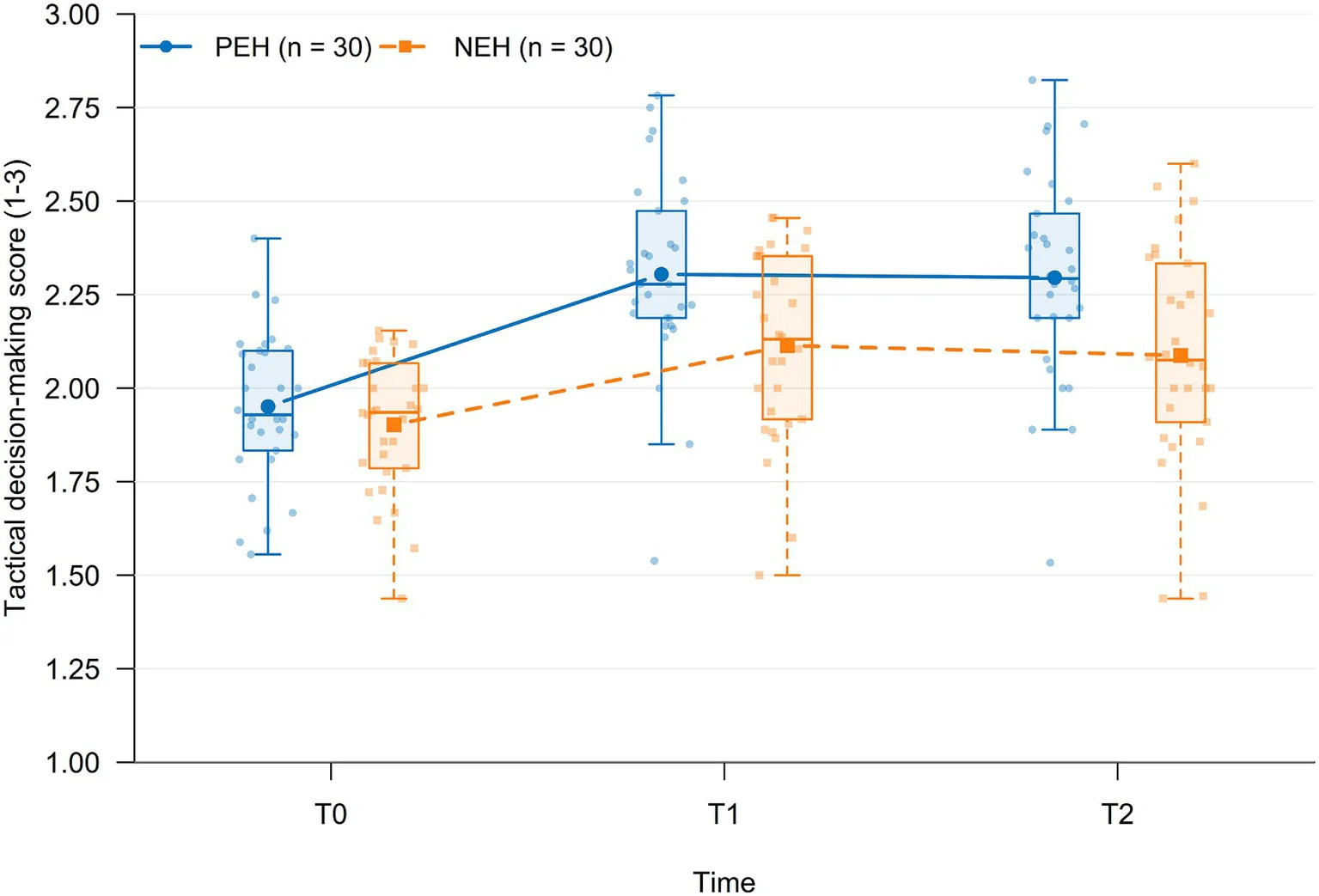
Distribution of tactical decision-making scores across T0, T1, and T2 by instructional condition.

**Table 5:** Tactical decision-making and perceived psychological safety by condition and time.

Outcome	Condition	T0 M (SD)	T1 M (SD)	T2 M (SD)
Decision score (1–3)	PEH	1.951 (0.202)	2.304 (0.258)	2.295 (0.279)
Decision score (1–3)	NEH	1.902 (0.179)	2.114 (0.251)	2.087 (0.291)
Psychological safety (1–5)	PEH	2.971 (0.488)	3.433 (0.459)	—
Psychological safety (1–5)	NEH	2.948 (0.667)	3.076 (0.871)	—

All 540 participant × wave × action-category combinations contained at least two eligible events. Thirty-one combinations (5.7%) contained exactly two events: 12 for dribbling/driving and 19 for shooting. Overall decision scores were based on 10–25 eligible events per participant per wave. Requiring at least three eligible events for the relevant action at every wave retained all 60 participants for passing, 49 for dribbling/driving (25 PEH, 24 NEH), and 43 for shooting (21 PEH, 22 NEH). The pattern of results was unchanged: the dribbling/driving interaction remained nonsignificant, *F*(1.606, 75.474) = 1.66, *p* = 0.202, ηp^2^ = 0.034, whereas the shooting interaction remained significant, *F*(2, 82) = 4.19, *p* = 0.019, ηp^2^ = 0.093. Passing results were unchanged because all participants met the criterion (Supplementary Table S3).

### 3.4 Perceived classroom psychological safety

The condition × time interaction for perceived classroom psychological safety was significant, *F*(1, 58) = 11.07, *p* = 0.002, ηp^2^ = 0.160. Psychological safety increased in PEH by 0.462 points (SE = 0.058), t(29) = 7.91, *p* < 0.001, whereas the NEH change of 0.129 points (SE = 0.081) was not statistically significant, t(29) = 1.58, *p* = 0.125.

### 3.5 Exploratory within-subject mediation analysis in the PEH condition

The exploratory mediation results for the PEH condition are summarized in Table 6. The estimated total effect of time on tactical decision-making was c = 0.354 (SE = 0.027; 95% CI [0.299, 0.408]), the direct effect controlling for change in psychological safety was c′ = 0.194 (SE = 0.033; 95% CI [0.127, 0.261]), and the model-implied indirect effect involving change in psychological safety was ab = 0.160 (bootstrap SE = 0.031; 95% bootstrap CI [0.100, 0.221]). Greater increases in perceived psychological safety covaried with greater improvements in tactical decision-making over the same T0-T1 interval. Because both changes were assessed over the same interval, this pattern should be interpreted as concurrent within-person covariation and does not establish temporal precedence or causal mediation. The corresponding exploratory within-subject mediation model is illustrated in Figure 4.

**Table 6:** Exploratory within-subject mediation estimates for the PEH condition.

Effect	Estimate	SE	95% CI / bootstrap CI
Total effect (c)	0.354	0.027	[0.299, 0.408]
Direct effect (c′)	0.194	0.033	[0.127, 0.261]
Indirect effect (ab)	0.16	0.031	[0.100, 0.221]
Effects are presented in the T1 − T0 direction for interpretive consistency. Signs from the original MEMORE output were reversed to correspond to this direction. Bootstrap confidence intervals are based on 5,000 resamples.			

**Figure 4:**
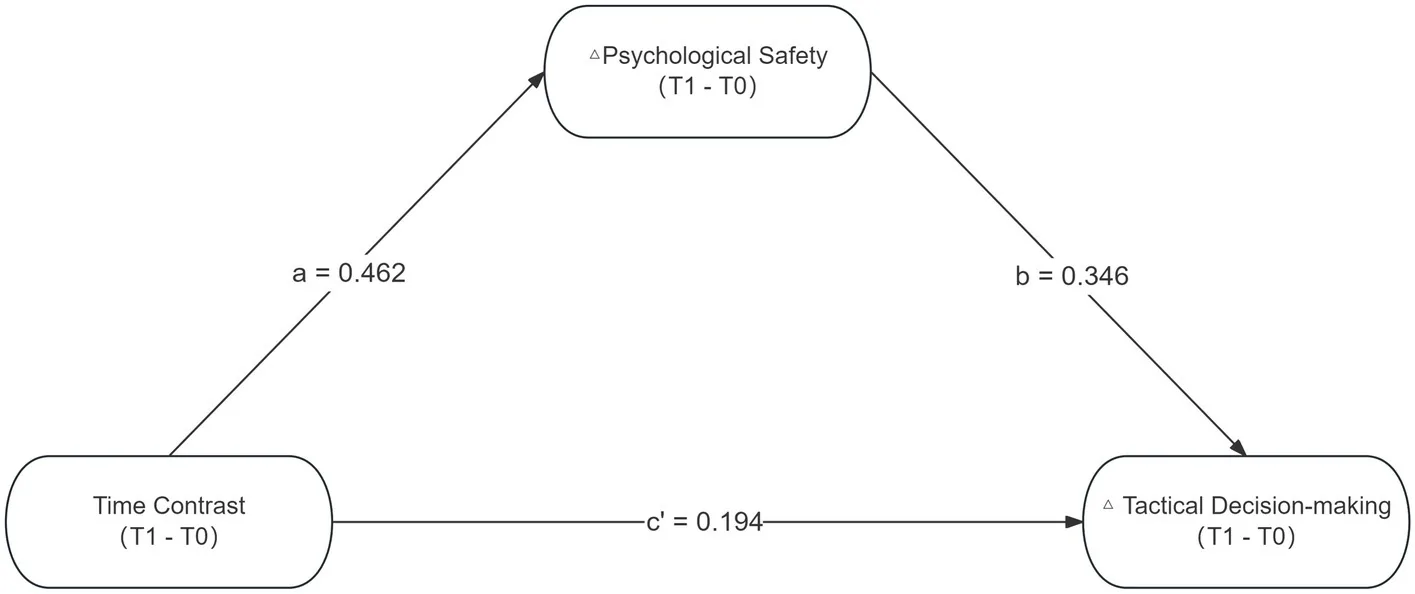
Exploratory within-subject mediation model for the PEH condition.

## 4 Discussion

### 4.1 Principal interpretation

The observed pattern supports the proposal that the social and instructional treatment of tactical errors matters even when both classes receive the same game-centered curriculum and the same initial tactical questions. Decision-making improved in both classes, but the PEH class showed a larger observed gain at posttest, and this advantage remained evident at the three-week retention assessment. This pattern is consistent with evidence that game-based approaches benefit decision-making, while suggesting one interpersonal explanation for heterogeneity across implementations (Ortiz et al., 2023; González-Valero et al., 2024; Manninen et al., 2025). The interpretation does not reduce TGfU to an affective intervention. Rather, the post-error response may support or constrain the cognitive work already required by TGfU: revealing perceived cues, comparing action alternatives, and re-entering play with a revised rule for action.

Improvement in both classes was theoretically coherent and was observed in the data. TGfU exposes learners to representative decision problems and provides repeated opportunities to coordinate perception and action; neutral error-handling delivered within TGfU therefore should not be expected to produce no learning. Basketball and wider youth-team-sport evidence supports gains from tactical curricula and representative practice even when the focal error protocol is absent (Pan et al., 2023; González-Valero et al., 2024; Manninen et al., 2025). The incremental contribution of PEH is that it may preserve engagement after visible mistakes and convert teacher feedback from a supplied answer into a short episode of self-explanation and guided reconstruction. The exploratory action-specific analyses showed significant condition × time interactions for passing and shooting, but not for dribbling/driving. Unequal decision opportunities warrant consideration because a fixed game duration does not ensure equal involvement, and means based on fewer events may be less precise or less representative of typical performance. However, the event-count sensitivity analysis preserved the original pattern after excluding participants with fewer than three eligible events for the relevant action at any wave. Dribbling/driving remained nonsignificant, whereas shooting remained significant despite the more restrictive sample. Thus, the action-specific findings were not materially altered by this particular restriction. Nevertheless, this sensitivity analysis does not establish equivalent score stability across participants or rule out effects of unequal decision opportunities, and eligible event counts do not capture off-ball engagement. Accordingly, the nonsignificant dribbling/driving interaction should not be attributed solely to limited decision opportunities or interpreted as evidence of no differential improvement.

### 4.2 Psychological safety and learning from tactical errors

The significant condition × time interaction in perceived psychological safety, together with the larger PEH pre-post increase, provides preliminary evidence extending classroom error-climate research into physical education. The public, embodied nature of basketball makes interpersonal risk especially salient: the learner cannot privately erase a poor decision, and the error may immediately affect teammates. Repeated teacher responses can therefore communicate whether error disclosure and retry are accepted forms of participation. This interpretation is compatible with recent psychological-safety reviews emphasizing learning behavior and leadership (Edmondson and Bransby, 2023), findings that constructive error climates promote adaptive reactions to mistakes (Soncini et al., 2022), and sport evidence linking psychological safety to team functioning and relationship quality (Jowett et al., 2023; Gosai et al., 2023; Vella et al., 2024).

The exploratory within-subject analysis in the PEH condition showed that larger increases in perceived psychological safety covaried with larger improvements in tactical decision-making over the same T0-T1 interval. This pattern is consistent with recent reviews linking psychological safety to learning behavior and performance (Edmondson and Bransby, 2023). However, the conclusion remains narrow. PEH is a multicomponent post-error package involving learner ownership, wait time, peer dialogue, and error framing; the model-implied indirect effect does not identify psychological safety as the sole explanatory process or isolate the contribution of any one component. Because psychological safety and tactical decision-making were assessed across the same two waves, the analysis does not establish that changes in psychological safety preceded changes in decision-making and should not be interpreted as evidence of temporal or causal mediation (Qin, 2024; Schuler et al., 2025). Stronger evidence would require temporally separated measurements of the proposed mediator and subsequent decision-making outcome, experimental manipulation of the mediator or individual protocol components, and replication across multiple instructional classes per condition.

### 4.3 Retention and practical significance

The gains observed at posttest were largely maintained in both conditions after 3 weeks without targeted instruction, while the PEH advantage remained evident at retention. This pattern is consistent with a more durable change in the learner’s decision rule, although the non-significant T1-T2 attenuation tests should not be treated as proof of perfect retention. Generating an explanation, comparing options, and replaying the action may create richer retrieval and correction processes than receiving the answer alone (Zhang and Fiorella, 2023; Metcalfe et al., 2025; Steuer et al., 2025b).

The protocol is deliberately modest for practice. Teachers do not need to praise every mistake or suspend performance standards. A usable sequence is to pause, allow a few seconds for reconstruction, ask what information was noticed, compare one or two options, and replay. The distinction between decision quality and technical outcome is essential (Aiyer et al., 2023): an open shot that misses can reflect good tactical judgment, whereas a contested shot that happens to score can remain a poor decision. Communicating this distinction may reduce outcome bias and make feedback more diagnostic.

### 4.4 Theoretical and methodological contributions

The study contributes by linking three levels of analysis that are often separated: an instructional model (TGfU), an observable teacher behavior (post-error handling), and a psychological mechanism (perceived safety). Methodologically, it distinguishes the common TGfU question sequence from the subsequent condition-specific response and assesses fidelity to both layers. It also treats decision-making as situated behavior rather than relying only on declarative tactical knowledge. The focused BALPAI adaptation reduces coding burden while retaining three on-ball offensive decision categories examined in subsequent applied BALPAI research (Rösch et al., 2022). Reporting action counts, action-specific scores, observer agreement, and the limits of the combined score also responds to evidence that BALPAI performance may depend on population and purpose (Rösch et al., 2022). The differing action-specific interaction patterns observed here further illustrate why the combined score should be interpreted alongside its component actions rather than as evidence of a uniform tactical effect. This pragmatic focus may be useful for physical education studies with limited observer resources, provided that authors do not present the three-action score as equivalent to the complete 11-action BALPAI.

### 4.5 Limitations and directions for future research

The main design limitation is that each condition was implemented in only one newly formed instructional class. Random allocation of individual students, gender stratification, a common curriculum, the same instructor, standardized feedback procedures, and fidelity checks reduce concern about systematic pre-existing group differences. However, individual randomization does not eliminate dependence that may emerge during instruction through shared peer interactions, class norms, schedules, and other contextual influences. With only one instructional class per condition, class-level dependencies and implementation-specific influences cannot be estimated separately from the condition effect. Accordingly, the present study is best regarded as a randomized instructional study with limited class-level replication rather than a cluster-randomized trial. Using the same instructor reduced between-teacher variability, but instructor blinding was not possible, and some expectancy or cross-condition carryover cannot be completely excluded despite condition-specific scripts and fidelity monitoring.

Second, the exploratory within-subject mediation analysis in the PEH condition included only 30 participants, limiting the precision of the estimated indirect effect; interpretation should therefore emphasize the bootstrap confidence interval. Third, psychological safety was self-reported and assessed at the same two waves as tactical decision-making. Accordingly, the analysis captures concurrent within-person change but cannot establish temporal precedence or causal mediation. Fourth, the classroom adaptation of Edmondson’s scale requires evidence of comprehension and internal consistency; with only two classes, class-level aggregation cannot be validated. Fifth, the focused BALPAI score omits off-ball and defensive decisions and may be influenced by how frequently each student handles the ball. Reporting action counts and action-specific results will help readers judge this concern. Sixth, PEH and NEH differ across several coordinated post-error components, so the study can estimate the effect of the package but cannot determine whether wait time, learner agency, peer dialogue, error framing, or their combination is responsible.

A stronger follow-up study should recruit multiple instructional classes per condition and use a cluster-randomized design, allowing hierarchical or multilevel models to account explicitly for students nested within classes and, where applicable, classes nested within teachers or schools. Psychological safety should be assessed during the instructional period and before a subsequent assessment of tactical decision-making to establish clearer temporal ordering; adaptive reactions to errors or participation could also be examined as more proximal mediators. Replication across gender, skill level, and educational stage would help clarify for whom positive error-handling is most beneficial.

## 5 Conclusion

This study suggests that the social and instructional treatment of tactical errors may matter alongside the tactical problems and questions used in game-centered basketball instruction. Both classes received the same TGfU inquiry sequence; only the post-error response differed. Embedding a brief, structured positive error-handling sequence within TGfU was associated with larger tactical decision-making gains that remained evident at retention. Within the PEH condition, greater increases in perceived classroom psychological safety covaried with greater pre-post improvements in tactical decision-making, suggesting a possible explanatory role for psychological safety. However, because these changes were assessed over the same T0-T1 interval, they do not establish temporal or causal mediation. The study provides an empirically informed first test using authentic 3-on-3 basketball assessment, while its one-instructional-class-per-condition implementation and multicomponent intervention require cautious interpretation and direct replication across multiple instructional classes.

## Data Availability

The raw data supporting the conclusions of this article will be made available by the authors, without undue reservation.
