# Supplementary material for: Positive error-handling in TGfU-based basketball instruction: effects on tactical decision-making and the role of perceived classroom psychological safety
Source: Front Psychol. 2026 Oct 16;17:1984425. doi: 10.3389/fpsyg.2026.1984425 (PMC13719377; doi:10.3389/fpsyg.2026.1984425)
Supplement: Supplementary file 1 [file Table_1.DOCX]

Supplementary Table S1. ANCOVA Sensitivity analyses of between-condition differences at T1 and T2

| **Wave** | **Covariate adjustment** | **PEH−NEH** | **95% CI** | **p** |
| --- | --- | --- | --- | --- |
| T1 | T0 decision score | 0.137 | [0.062, 0.211] | <0.001 |
| T1 | T0 decision score + age + gender | 0.136 | [0.059, 0.212] | <0.001 |
| T2 | T0 decision score | 0.152 | [0.056, 0.249] | 0.003 |
| T2 | T0 decision score + age + gender | 0.153 | [0.054, 0.252] | 0.003 |
| ***Note.*** *Positive PEH − NEH estimates indicate higher adjusted decision scores in the PEH condition.* | | | | |

Supplementary Table S2. Action-specific tactical decision-making scores and eligible action counts by condition and time

| **Action** | **Condition** | **T0 M (SD)** | **T0 events** | **T1 M (SD)** | **T1 events** | **T2 M (SD)** | **T2 events** | **Condition × Time** | **ηp²** |
| --- | --- | --- | --- | --- | --- | --- | --- | --- | --- |
| Passing | PEH | 1.983 (0.208) | 228 | 2.348 (0.257) | 249 | 2.342 (0.281) | 236 | F(1.700, 98.580) = 10.25; p < 0.001 | 0.15 |
|  | NEH | 1.938 (0.188) | 222 | 2.152 (0.261) | 213 | 2.114 (0.312) | 224 |  |  |
| Dribbling/driving | PEH | 1.974 (0.214) | 144 | 2.300 (0.296) | 160 | 2.256 (0.318) | 158 | F(1.814, 105.227) = 1.55; p = 0.218 | 0.026 |
|  | NEH | 1.893 (0.185) | 133 | 2.151 (0.263) | 154 | 2.080 (0.268) | 152 |  |  |
| Shooting | PEH | 1.877 (0.229) | 135 | 2.247 (0.281) | 134 | 2.237 (0.271) | 126 | F(2, 116) = 7.17; p = 0.001 | 0.11 |
|  | NEH | 1.837 (0.224) | 112 | 2.028 (0.252) | 138 | 2.029 (0.317) | 121 |  |  |
| ***Note.*** *Scores are participant-level means of all eligible FinalScore ratings (1 = inadequate, 2 = neutral/ambiguous, 3 = adequate) for the specified action. Event counts are total eligible action events across the 30 participants in each condition at each time point. Condition × Time effects are from separate 2 × 3 mixed ANOVAs for each action. Passing violated sphericity (Mauchly W = 0.823, p = 0.004; ε = 0.850), as did dribbling/driving (W = 0.898, p = 0.046; ε = 0.907); Greenhouse–Geisser corrections were therefore applied. Sphericity was not violated for shooting (W = 0.926, p = 0.111).* | | | | | | | | | |

Supplementary Table S3. Sensitivity analyses of action-specific tactical decision-making after excluding participants with fewer than three eligible events at any assessment wave

| **Action** | **Analysis sample** | **PEH, n** | **NEH, n** | **Condition × time** | **p** | **ηp²** |
| --- | --- | --- | --- | --- | --- | --- |
| Passing | Full sample | 30 | 30 | F(1.700, 98.580) = 10.25 | < 0.001 | 0.15 |
|  | Restricted sample | 30 | 30 | F(1.700, 98.580) = 10.25 | < 0.001 | 0.15 |
| Dribbling/driving | Full sample | 30 | 30 | F(1.814, 105.227) = 1.55 | 0.218 | 0.026 |
|  | Restricted sample | 25 | 24 | F(1.606, 75.474) = 1.66 | 0.202 | 0.034 |
| Shooting | Full sample | 30 | 30 | F(2, 116) = 7.17 | 0.001 | 0.11 |
|  | Restricted sample | 21 | 22 | F(2, 82) = 4.19 | 0.019 | 0.093 |
| ***Note.*** *PEH = positive error-handling; NEH = neutral error-handling; ηp² = partial eta squared. For each action, the restricted sample included only participants with at least three eligible coded decision events for that action at each assessment wave (T0, T1, and T2). Eligibility was determined separately for each action. Scores were participant-level means of eligible event ratings. Separate 2 × 3 mixed ANOVAs tested condition × time interactions. Greenhouse–Geisser corrections were applied when sphericity was violated. The three-event threshold was introduced during revision as an exploratory sensitivity criterion, not as a validated minimum for reliable individual scoring. The full-sample analyses remained the primary analyses.* | | | | | | |

Supplementary Table S4. One-factor CFA fit for the adapted psychological-safety scale

| **Fit statistic** | **χ²/DF** | **CFI** | **TLI** | **RMSEA** | **SRMR** |
| --- | --- | --- | --- | --- | --- |
| T0 | 1.329 | 0.992 | 0.988 | 0.075 | 0.061 |
| T1 | 1.493 | 0.993 | 0.99 | 0.091 | 0.048 |
